# Dysregulation of lncRNAs in circulation of patients with periodontitis: results of a pilot study

**DOI:** 10.1186/s12903-021-01851-2

**Published:** 2021-09-25

**Authors:** Soudeh Ghafouri-Fard, Leila Gholami, Elham Badrlou, Saba Sadeghpour, Naghme Nazer, Mahdi Shadnoush, Arezou Sayad, Mohammad Taheri

**Affiliations:** 1 Department of Medical Genetics, Shahid Beheshti University of Medical Sciences, Tehran, Iran; 2 Department of Periodontics, Dental Research Center, Hamadan University of Medical Sciences, Hamadan, Iran; 3 Pediatric Cell and Gene Therapy Research Center, Tehran University of Medical Sciences, Tehran, Iran; 4 Hematopoietic Stem Cell Research Center, Shahid Beheshti University of Medical Sciences, Tehran, Iran; 5 Department of Electrical Engineering, Sharif University of Technology, Tehran, Iran; 6 Department of Clinical Nutrition, Faculty of Nutrition and Food Technology, Shahid Beheshti University of Medical Sciences, Tehran, Iran; 7 Dental Research Center, Research Institute for Dental Sciences, Dental School, Shahid Beheshti University of Medical Sciences, Tehran, Iran; 8 Skull Base Research Center, Loghman Hakim Hospital, Shahid Beheshti University of Medical Sciences, Tehran, Iran

**Keywords:** RNA, Long noncoding, Periodontitis

## Abstract

**Background:**

Periodontitis is a chronic inflammatory disorder with a complex etiology. Long non-coding RNAs (lncRNAs) have been shown to affect pathoetiology of periodontitis. We aimed at identification of expression of five lncRNAs, namely *Linc0116, Linc00667, CDK6-AS1, FENDRR* and *DIRC3* in the circulation and gingival tissues of these patients compared with healthy controls.

**Methods:**

In a pilot case–control study, we compared expressions of *Linc0116, Linc00667, CDK6-AS1, FENDRR* and *DIRC3* lncRNAs between blood and tissue samples of patients with periodontitis and healthy controls using real time quantitative PCR technique. The present work was performed on samples got from 26 patients with periodontitis and 28 controls. Female/male ratio was 16/10 and 12/16 in cases and controls, respectively.

**Results:**

There was no significant difference in the expressions of *Linc0116, Linc00667, CDK6-AS1, FENDRR* and *DIRC3* genes between affected and unaffected tissues. However, expressions of *Linc0116, Linc00667, CDK6-AS1, FENDRR* and *DIRC3* genes were significantly lower in the blood samples of patients when compared with control samples (Ratio of mean expression = 0.16, 0.14, 0.13, 0.10 and 0.14, respectively). Subsequently, we compared expressions of these lncRNAs between patients and controls in a sex-based manner. Expressions of *Linc00667, FENDRR* and *DIRC3* genes were significantly lower in female patients compared with female controls (RME = 0.09, 0.07 and 0.10, respectively). Yet, there was no significant difference in expression of any of mentioned lncRNAs among male subgroups. Consistent with the similar levels of *Linc0116, Linc00667, CDK6-AS1, FENDRR* and *DIRC3* in tissue samples of patients and controls, none of them could separate these two sets of samples. However, AUC values for of *Linc0116, Linc00667, CDK6-AS1, FENDRR* and *DIRC3* expression levels in blood samples were 0.66, 0.72, 0.70, 0.72, 0.70 and 0.68, respectively with *FENDRR* having the best sensitivity value.

**Conclusion:**

Taken together, lncRNAs might be involved in the pathologic events in the circulation of patients with periodontitis.

## Background

Long non-coding RNAs (lncRNAs) are transcripts with sizes more than 200 nucleotides and diverse regulatory roles on gene expression. Through acting as coactivators of transcription factors, they can modulate expression of numerous genes [1]. Moreover, lncRNAs can affect different layers of gene expression through modulating chromatin arrangement, affecting epigenetic mechanisms, regulating RNA stability and interacting with several biomolecules [2]. These transcripts influence the pathoetiology of several disorders, including periodontitis [3]. Several lncRNAs have been found to be dysregulated in the circulation, gingival tissues, primary human gingival fibroblasts or periodontal ligament cells of patients with periodontitis [3]. These lncRNAs affect the pathogenic course of periodontitis through influencing cell apoptosis [4], autophagy [5], immune responses [6] and osteogenic differentiation [7]. Although the impact of numerous lncRNAs in the pathogenesis of periodontitis has been assessed, the role of several other lncRNAs in this condition remained to be elucidated. We aimed at identification of expression of five lncRNAs, namely *Linc0116, Linc00667, CDK6-AS1, FENDRR* and *DIRC3* in the circulation and gingival tissues of these patients compared with healthy controls.

*Linc00667* is a long intergenic RNA whose function has not been completely understood. *Linc01116* is a relatively newly identified lncRNA whose expression has been found to be increased in prostate cancer [8], non-small cell lung cancer [9], and glioma [10]. In the latter type of cancer, it can promote neutrophil recruitment through DDX5-associated modulation of IL-1β [11]. *CDK6-AS1* is another lncRNA that regulate cell migration and invasion in asynergic manner with CDK6 [12]. *FENDRR* gene has been shown to produce a spliced lncRNA transcribed bidirectionally with FOXF1. The mouse homologue of this gene has a crucial role in the developmental processes through binding with PRC2 and/or TrxG/MLL complexes and enhancing methylation of the target genes promoters [13]. Finally, *DIRC3* is a MITF-SOX10 regulated gene that suppresses cell proliferation and anchorage-independent growth. This lncRNA is primarily located in the nucleus where it activates its nearby gene i.e. *IGFBP5* via changing chromatin configuration [14]. We hypothesized that expression levels of these lncRNAs are different between healthy controls and affected persons, so that they can be used as markers for this condition.

## Methods

### Enrolled persons

The present work was performed on samples got from 26 patients with periodontitis and 28 controls. Tissue specimens were obtained from patients suffered from chronic periodontitis, based on the criteria described in our former study [15]. Patients had stages III (with remarkable impairment of the attachment apparatus) or IV (noticeable injury of the periodontal support which might lead to significant tooth loss and defects in the masticatory function) of periodontitis [16]. Smoking, history of inflammatory disorders, malignancies, diabetes and intake of antibiotics or anti-inflammatory agents were regarded as exclusion criteria. All samples were obtained from the periodontal clinics of Hamadan University of Medical Sciences. Control samples were excised from individuals in the course of dental crown lengthening surgery. These individuals had no signs of periodontitis. The clinical variables were assessed by a periodontics. Sociodemographic data were recorded in a questionnaire. Informed consents were obtained from all study participants. The study protocol was approved by the ethical committee of Shahid Beheshti University of Medical Sciences.

### Expression assay

Expression of genes were compared between groups using quantitative real time PCR method. For this purpose, RNA was isolated from tissues and venous blood samples using PicoPure™ RNA Isolation Kit (Thermo Fisher Scientific). Complementary DNA was produced from these specimens by using the Smobio cDNA production kit (Taiwan). Expression amounts of *Linc0116, Linc00667, CDK6-AS1, FENDRR* and *DIRC3* lncRNAs were quantified in tissues and blood samples using the qRT-PCR kit (GeneDireX, Taiwan). Thermal cycling reactions were accomplished in LightCycler® 96 instrument. Real time PCR program included a primary activation period at 95 °C for ten minutes. Then, the program included 40 cycles of denaturation at 95 °C for 10 s and annealing/extension at 65 °C for 40 s. All reactions were performed in duplicate. Primer sequences are demonstrated in Table 1.

**Table 1:** Primer sequences

Gene name	Sequence
*HPRT1*	F: AGCCTAAGATGAGAGTTC R: CACAGAACTAGAACATTGATA
*Linc0116*	F: AACGCTTTTGAATATGGGGAC R: CAATCACAGAGCTCTCCTTGC
*Linc00667*	F: AATTGGAAGGAAACACAGCC R: GACTGCAGGCCACAGACAG
*CDK6-AS1*	F: CACGGCGTGGCAGCTTTCAG R: AGCAGCAAAGCAAAGCCTGGGA
*FENDRR*	F: TAAAATTGCAGATCCTCCCG R: AACGTTCGCATTGGTTTAGC
*DIRC3*	F: GGGAGTATGCCTCCAGACAG R: GTCGATCAGCAAGCTCAGTG

### Statistical analyses

Statistical variables were studied using R programming language. Expressions of *Linc0116, Linc00667, CDK6-AS1, FENDRR* and *DIRC3* transcripts were measured using Ct and PCR efficiency values after log-transforming of the raw data. Mean values of genes expressions were compared between patients and healthy persons using t-test. Spearman correlation coefficient was measured to estimate correlations between expressions of *Linc0116, Linc00667, CDK6-AS1, FENDRR* and *DIRC3* genes. Diagnostic power of *Linc0116, Linc00667, CDK6-AS1, FENDRR* and *DIRC3* genes was assessed via illustrating receiver operating characteristic curves and calculation of the area under these curves (AUC).

## Results

The present work was performed on samples got from 26 patients with periodontitis and 28 controls. Female/male ratio was 16/10 and 12/16 in cases and controls, respectively. Mean age (± standard deviation) was 37.6 ± 2.5 and 37.5 ± 1.7 in cases and controls, respectively.

### Expression assays

**Fig. 1:**
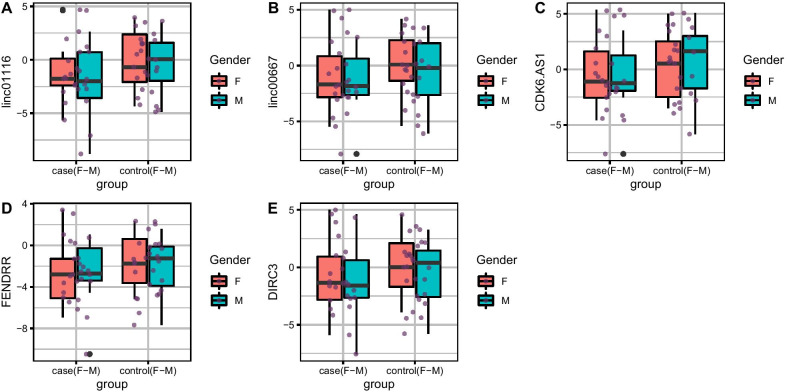
Relative expression amounts of *Linc0116, Linc00667, CDK6-AS1, FENDRR* and *DIRC3* genes in affected tissues compared with control tissues

**Fig. 2:**
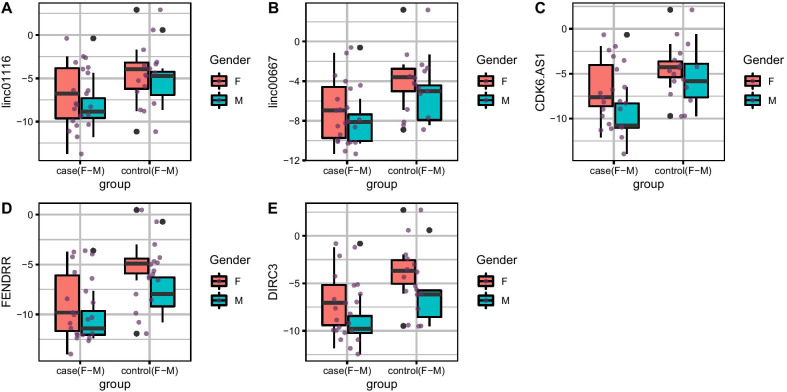
Relative expression amounts of *Linc0116, Linc00667, CDK6-AS1, FENDRR* and DIRC3 genes in blood samples of patients with periodontitis compared with controls

Since there was no previous data about the expression levels of mentioned genes in the periodontitis, a previous sample size calculation was not performed and the current study was a pilot study. Relative expressions of *Linc0116, Linc00667, CDK6-AS1, FENDRR* and *DIRC3* genes in tissues and blood specimens of patients and healthy subjects are depicted in Figs. 1 and 2, respectively.

There was no significant difference in the expressions of *Linc0116, Linc00667, CDK6-AS1, FENDRR* and *DIRC3* genes between affected and unaffected tissues. However, expressions of *Linc0116, Linc00667, CDK6-AS1, FENDRR* and *DIRC3* genes were significantly lower in the blood samples of patients when compared with control samples (Ratio of mean expression = 0.16, 0.14, 0.13, 0.10 and 0.14, respectively). Subsequently, we compared expressions of these lncRNAs between patients and controls in a sex-based manner. Expressions of *Linc00667, FENDRR* and *DIRC3* genes were significantly lower in female patients compared with female controls (RME = 0.09, 0.07 and 0.10, respectively). Yet, there was no significant difference in expression of any of mentioned lncRNAs among male subgroups (Table 2).

**Table 2:** Statistical parameters of assessment of expression of *Linc0116, Linc00667, CDK6-AS1, FENDRR* and *DIRC3* genes in tissues and blood specimens gathered from patients compared with controls (*RME* ratio of mean expression)

	*Linc0116*					*Linc00667*					*CDK6-AS1*					*FENDRR*					*DIRC3*				
Number of Samples	SE	RME	*P* value	95% CI		SE	RME	*P* value	95% CI		SE	RME	*P* value	95% CI		SE	RME	*P* value	95% CI		SE	RME	*P* value	95% CI	
*Tissues*																									
Total	0.81	0.44	0.14	− 2.81	0.43	0.83	0.63	0.42	− 2.35	1.00	0.89	0.51	0.28	− 2.75	0.80	0.79	0.60	0.36	− 2.33	0.86	0.81	0.63	0.42	− 2.30	0.97
F	1.12	0.47	0.34	− 3.43	1.25	1.22	0.58	0.53	− 3.30	1.74	1.27	0.70	0.69	− 3.15	2.11	1.13	0.48	0.37	− 3.39	1.30	1.15	0.54	0.45	− 3.26	1.50
M	1.35	0.32	0.24	− 4.53	1.23	1.20	0.55	0.48	− 3.36	1.66	1.29	0.35	0.25	− 4.21	1.18	1.24	0.67	0.65	− 3.19	2.05	1.27	0.64	0.62	− 3.32	2.04
*Blood*																									
Total	1.11	0.16	0.02	− 4.86	− 0.37	0.97	0.14	0.01	− 4.78	− 0.84	1.06	0.13	0.01	− 5.06	− 0.77	1.04	0.10	0.00	− 5.47	− 1.24	1.07	0.14	0.01	− 5.04	− 0.68
F	1.51	0.17	0.10	− 5.77	0.59	1.28	0.09	0.02	− 6.10	− 0.74	1.27	0.16	0.05	− 5.32	− 0.03	1.31	0.07	0.01	− 6.53	− 1.06	1.27	0.10	0.02	− 6.03	− 0.70
M	1.73	0.15	0.14	− 6.50	0.98	1.50	0.25	0.21	− 5.23	1.24	1.86	0.08	0.07	− 7.65	0.40	1.66	0.13	0.10	− 6.50	0.69	1.82	0.19	0.21	− 6.33	1.54

**Fig. 3:**
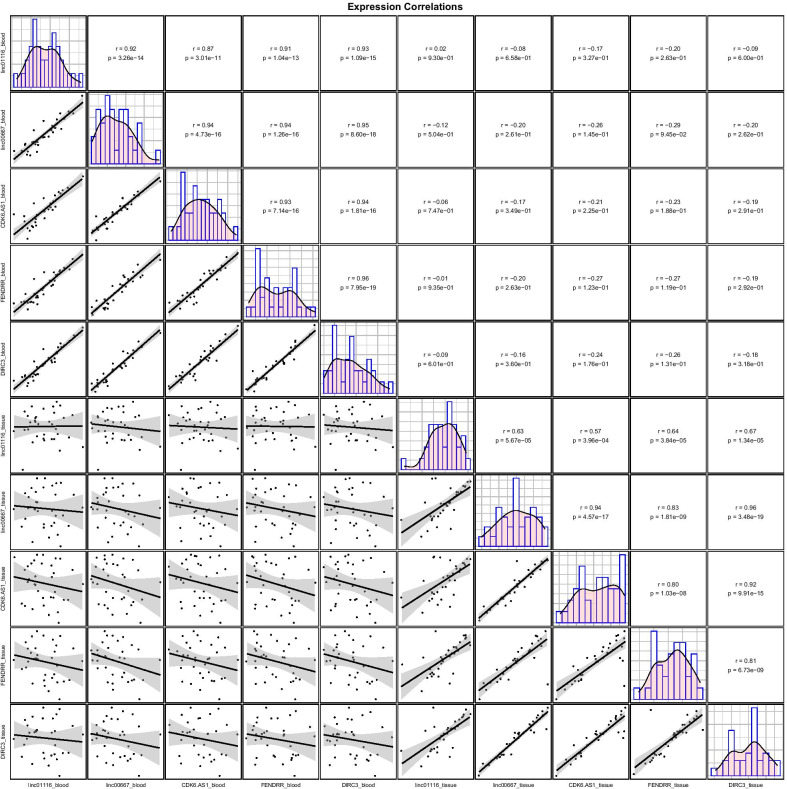
Correlations between tissue/ blood levels of *Linc0116, Linc00667, CDK6-AS1, FENDRR* and *DIRC3* genes. Distributions of parameters is shown on the diagonals. Bivariate scatter plots are depicted on the lower divisions for each pair. Correlation coefficients and *P* values are demonstrated on the upper parts

Expressions of *Linc0116, Linc00667, CDK6-AS1, FENDRR* and *DIRC3* genes were robustly correlated with each other in the blood samples with r values ranging from 0.87 to 0.94. Similarly, their tissues levels were correlated with each other with r values ranging from 0.81 to 0.96. Yet, there was no significant correlation between expression levels of an individual lncRNA in blood and tissue samples (Fig. 3).

**Fig. 4:**
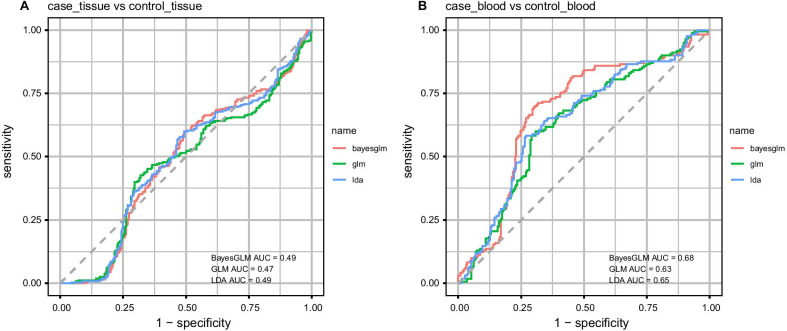
ROC curves depicted using the Bayesian Generalized Linear Model. Consistent with the similar levels of *Linc0116, Linc00667, CDK6-AS1, FENDRR* and *DIRC3* in tissue samples of patients and controls, none of them could separate these two sets of samples

In order to assess the diagnostic power of *Linc0116, Linc00667, CDK6-AS1, FENDRR* and *DIRC3* genes in blood and tissue samples, we depicted ROC curves using the Bayesian Generalized Linear Model (Fig. 4).

However, AUC values for of *Linc0116, Linc00667, CDK6-AS1, FENDRR* and *DIRC3* expression levels in blood samples were 0.66, 0.72, 0.70, 0.72, 0.70 and 0.68, respectively with *FENDRR* having the best sensitivity value (Table 3).

**Table 3:** Statistical parameters of ROC curves in tissue and blood samples

	*Linc0116*			*Linc00667*			*CDK6-AS1*			*FENDRR*			*DIRC3*			All		
Number of Samples	AUC	Sensitivity	Specificity	AUC	Sensitivity	Specificity	AUC	Sensitivity	Specificity	AUC	Sensitivity	Specificity	AUC	Sensitivity	Specificity	AUC	Sensitivity	Specificity
Tissues	0.58	0.64	0.62	0.54	0.57	0.66	0.55	0.57	0.67	0.54	0.60	0.59	0.53	0.57	0.64	0.49	0.60	0.51
Blood samples	0.66	0.69	0.70	0.72	0.71	0.73	0.70	0.73	0.72	0.72	0.81	0.65	0.70	0.76	0.69	0.68	0.70	0.70

## Discussion

LncRNAs have crucial roles in several aspects of biological processes in the course of periodontitis [3]. As regulation of cell proliferation/apoptosis and modulation of immune reactions are important functions of lncRNAs, several lncRNAs are involved in both oncogenic processes and immune-related conditions such as periodontitis [3]. In the current pilot study, we assessed expressions of a number of lncRNAs with known functions in the carcinogenesis in the biological samples obtained from patients with periodontitis.

While we detected no significant difference in the expressions of *Linc0116, Linc00667, CDK6-AS1, FENDRR* and *DIRC3* genes between affected and unaffected tissues, expressions of all assessed lncRNAs were significantly lower in the blood samples of patients when compared with control samples. Assessment of blood levels of lncRNAs has an important application in the clinical settings as this type of sampling facilitates patients’ follow-up in a non-invasive manner. A previous study in patients with periodontitis has indicated over-expression of the lncRNA *AWPPH* in patients who experienced disease recurrence but not in patients without recurrence [17], indicating the appropriateness of this lncRNA for patients’ follow-up. We have recently reported down-regulation of *ANRIL* lncRNA in the circulation of patients with periodontitis [15]. It is worth mentioning that both *AWPPH* and *ANRIL* are among lncRNAs with crucial roles in the carcinogenic processes. These observations further emphasize on the similarities in cellular processes which are affected by lncRNAs in cancers and periodontitis.

Subsequently, we compared expressions of these lncRNAs between patients and controls in a sex-based manner. Expressions of *Linc00667, FENDRR* and *DIRC3* genes were significantly lower in female patients compared with female controls (RME = 0.09, 0.07 and 0.10, respectively). Yet, there was no significant difference in expression of any of mentioned lncRNAs among male subgroups. The prevalence of periodontitis has is remarkably higher males compared to females, suggesting a potential sex-based bias in pathoetiology of this condition [18]. A previous study that assessed risk factors for periodontal diseases has demonstrated the role of gender in conferring risk of this condition [19]. Moreover, when the analysis was restricted to patients with severe periodontitis, male subjects were found to be at higher risk compared to females [19]. The underlying mechanism of sex-based dysregulation of mentioned lncRNAs and their potential interaction with risk factors for periodontitis should be uncovered in future studies.

Next, we examined the correlations between expression levels of *Linc0116, Linc00667, CDK6-AS1, FENDRR* and *DIRC3* in both sets of samples. Notably, expressions of these lncRNAs were robustly correlated with each other in both blood samples and gingival tissues. This finding might suggest interaction between these lncRNAs and presence of a single regulatory mechanism for controlling expression of these lncRNAs. Yet, there was no significant correlation between expression levels of an individual lncRNA in blood and tissue samples. Therefore, expression levels of these lncRNAs might be under tissue-dependent regulatory mechanisms.

Consistent with the similar levels of *Linc0116, Linc00667, CDK6-AS1, FENDRR* and *DIRC3* in tissue samples of patients and controls, none of them could separate these two sets of samples. However, AUC values for of these lncRNAs, particularly *Linc00667* and *FENDRR* were appropriate.

Our study had limitation in terms of sample size and lack of age and gender-pairing. Moreover, we did not assess expression of these lncRNAs in other oral pathologic conditions to appraise the specificity of these lncRNAs for periodontitis. Finally, we did not have the detailed clinicopathological data such as mean clinical attachment level or mean pocket depth.

## Conclusion

Taken together, lncRNAs might be involved in the pathologic events in the circulation of patients with periodontitis. Future assessment of expression of these lncRNAs during different stages of periodontitis is required for evaluation of their impact on disease progression.

## Data Availability

The datasets used and/or analyzed during the current study are available from the corresponding author on reasonable request.

## Abbreviations

lncRNAs: Long non-coding RNAs
AUC: Area under curves
ROC: Receiver operating characteristic
CDK6-AS1: CDK6 antisense RNA 1
FENDRR: FOXF1 adjacent non-coding developmental regulatory RNA
DIRC3: Disrupted in renal carcinoma 3

## References

[1] Hu G, Gong A-Y, Wang Y, Ma S, Chen X, Chen J (2016). LincRNA-Cox2 promotes late inflammatory gene transcription in macrophages through modulating SWI/SNF-mediated chromatin remodeling. J Immunol.

[2] Wang KC, Chang HY (2011). Molecular mechanisms of long noncoding RNAs. Mol Cell.

[3] Sayad A, Mirzajani S, Gholami L, Razzaghi P, Ghafouri-Fard S, Taheri M (2020). Emerging role of long non-coding RNAs in the pathogenesis of periodontitis. Biomed Pharmacother.

[4] Shi B, Shao B, Yang C, Guo Y, Fu X, Gan N (2019). Upregulation of JHDM1D-AS1 protects PDLSCs from H2O2-induced apoptosis by decreasing DNAJC10 via phosphorylation of eIF2α. Biochimie.

[5] Guo R, Huang Y, Liu H, Zheng Y, Jia L, Li W (2020). Long non-coding RNA H19 participates in periodontal inflammation via activation of autophagy. J Inflamm Res.

[6] Bochenek G, Häsler R, El Mokhtari N-E, König IR, Loos BG, Jepsen S (2013). The large non-coding RNA ANRIL, which is associated with atherosclerosis, periodontitis and several forms of cancer, regulates ADIPOR1, VAMP3 and C11ORF10. Hum Mol Genet.

[7] Xu Y, Qin W, Guo D, Liu J, Zhang M, Jin Z. LncRNA-TWIST1 promoted osteogenic differentiation both in PPDLSCs and in HPDLSCs by inhibiting TWIST1 expression. BioMed Res Int. 2019;2019. 10.1155/2019/8735952 PMC6612385 31341908

[8] Beaver LM, Kuintzle R, Buchanan A, Wiley MW, Glasser ST, Wong CP (2017). Long noncoding RNAs and sulforaphane: a target for chemoprevention and suppression of prostate cancer. J Nutr Biochem.

[9] Liang Y, Ma Y, Li L, Shen X, Xin T, Zhao Y (2018). Effect of long non-coding RNA LINC01116 on biological behaviors of non-small cell lung cancer cells via the hippo signaling pathway. J Cell Biochem.

[10] Zhang N, Shuai K, Cheng J, Yang W, Kan Z (2019). LncRNA linc01116 prometes glioma cell migration and invasion by modulation of radixin targeted by miR-31. Int J Clin Exp Pathol.

[11] Wang T, Cao L, Dong X, Wu F, De W, Huang L (2020). LINC01116 promotes tumor proliferation and neutrophil recruitment via DDX5-mediated regulation of IL-1β in glioma cell. Cell Death Dis.

[12] Liu J, Wang Y, Chen P, Ma Y, Wang S, Tian Y (2019). AC002454.1 and CDK6 synergistically promote endometrial cell migration and invasion in endometriosis. Reproduction (Cambridge, England).

[13] Grote P, Wittler L, Hendrix D, Koch F, Währisch S, Beisaw A (2013). The tissue-specific lncRNA Fendrr is an essential regulator of heart and body wall development in the mouse. Dev Cell.

[14] Coe EA, Tan JY, Shapiro M, Louphrasitthiphol P, Bassett AR, Marques AC (2019). The MITF-SOX10 regulated long non-coding RNA DIRC3 is a melanoma tumour suppressor. PLoS Genet.

[15] Gholami L, Ghafouri-Fard S, Mirzajani S, Arsang-Jang S, Taheri M, Dehbani Z (2020). The lncRNA ANRIL is down-regulated in peripheral blood of patients with periodontitis. Noncoding RNA Res.

[16] Tonetti MS, Greenwell H, Kornman KS (2018). Staging and grading of periodontitis: framework and proposal of a new classification and case definition. J Periodontol.

[17] Wang X, Ma F, Jia P (2019). LncRNA AWPPH overexpression predicts the recurrence of periodontitis. Biosci Rep.

[18] Ioannidou E (2017). The sex and gender intersection in chronic periodontitis. Front Public Health.

[19] Eke PI, Wei L, Thornton-Evans GO, Borrell LN, Borgnakke WS, Dye B (2016). Risk indicators for periodontitis in US adults: NHANES 2009 to 2012. J Periodontol.

